# Implications of Glycosylation in Alzheimer’s Disease

**DOI:** 10.3389/fnins.2020.625348

**Published:** 2021-01-13

**Authors:** Henriette Haukedal, Kristine K. Freude

**Affiliations:** Department of Veterinary and Animal Sciences, Faculty of Health and Medical Sciences, University of Copenhagen, Frederiksberg, Denmark

**Keywords:** Alzheimer’s disease, glycans, neuroinflammation, AD biomarkers, AD therapeutics, iPSCs

## Abstract

Alzheimer’s disease (AD) is the most common cause of dementia, affecting millions of people worldwide, and no cure is currently available. The major pathological hallmarks of AD are considered to be amyloid beta plaques and neurofibrillary tangles, generated by respectively APP processing and Tau phosphorylation. Recent evidence imply that glycosylation of these proteins, and a number of other AD-related molecules is altered in AD, suggesting a potential implication of this process in disease pathology. In this review we summarize the understanding of glycans in AD pathogenesis, and discuss how glycobiology can contribute to early diagnosis and treatment of AD, serving as potential biomarkers and therapeutic targets. Furthermore, we look into the potential link between the emerging topic neuroinflammation and glycosylation, combining two interesting, and until recent years, understudied topics in the scope of AD. Lastly, we discuss how new model platforms such as induced pluripotent stem cells can be exploited and contribute to a better understanding of a rather unexplored area in AD.

## Introduction

### Alzheimer’s Disease

Life expectancy has been steadily increasing over the past 200 years, first due to improvements within housing, sanitation and education, followed by the revolutionary development of vaccines and antibiotics. Although these improvements have significantly reduced the early/mid-life mortality, together with an exponentially aging population we are facing new health challenges (American Association for the Advancement of Science, 2008). More than 50 million people worldwide are affected by dementia, and the numbers are expected to reach 150 million in 2050, making it not only a global health concern, but also an economical burden. Approximately 70% of all dementia cases are caused by Alzheimer’s disease (AD), making it the most prevalent of all neurodegenerative disorders (Alzheimer’s Association, 2018). AD is characterized by an irreversible loss of neurons, particularly in the cortex and hippocampus, causing a progressive decline in memory. There is currently no cure for AD, and the disease typically runs its course over 5–10 years after symptoms appear. However, it is believed that AD initiated in the brain decades before clinical symptoms appear and a diagnosis can be made, making timely medical intervention substantially more challenging. Research within the area is therefore crucial to develop new early-on diagnostic tools and potential new therapeutic approaches.

**FIGURE 1:**
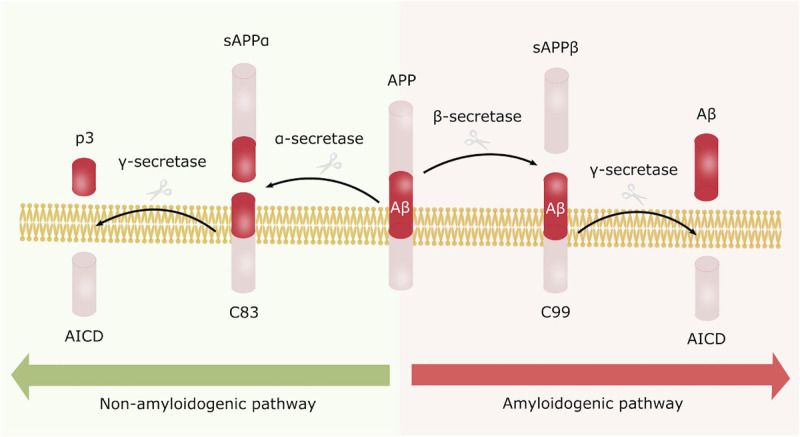
Amyloid precursor protein processing. APP can be processed either through the non-amyloidogenic **(left)** or the amyloidogenic pathway **(right)**. In the non-amyloidogenic processing pathway, APP is initially cleaved by α-secretase generating sAPPα and C83. C83 is further processed by γ-secretase producing AICD and the non-toxic p3 fragment. In contrary, APP is first processed by β-secretase in the amyloidogenic pathway, leading to formation of sAPPβ and C99, where C99 is further cleaved by γ-secretase generating AICD and Aβ peptides. These peptides are prone to aggregate into the toxic Aβ plaques, characteristic for AD.

**FIGURE 2:**
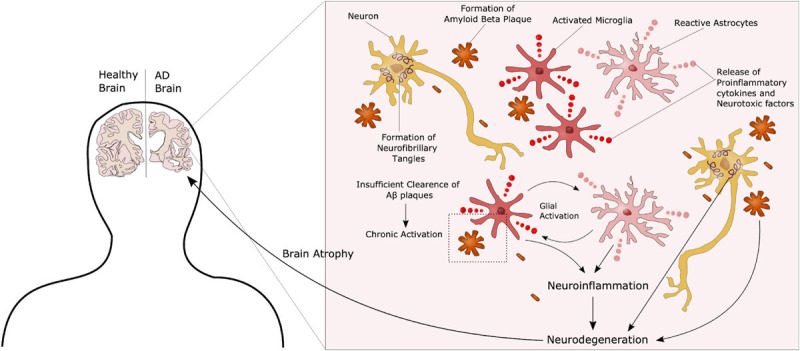
Complex AD Pathology. AD is characterized by the formation of extracellular amyloid beta plaques and intracellular Tau tangles. Additionally, chronic activation of microglia due to an inability to clear such plaques through phagocytosis, leads to a state of neuroinflammation in these patients, further contributing to disease progression. These neuropathological hallmarks ultimately lead to neurodegeneration and atrophy of the brain.

Alzheimer’s disease can be categorized as familial (fAD) or sporadic (sAD). sAD is by far the most common form of AD, accounting for approximately 95% of all cases, and is known to be a multifactorial disease where genetic risk factors in combination with adverse environmental factors play a role in disease development. Although age is recognized as the most common non-modifiable risk factor, genetic factors such as the ε4 allele of Apolipoprotein E (*APOE4*) are known to increase the risk of developing sAD. More recent, genetic profiles related to the innate immune system have been shown to increase the risk of sAD, emphasizing the potential role of non-neuronal cells and neuroinflammation in neurodegenerative disorders (Efthymiou and Goate, 2017). fAD on the other hand, is caused by specific mutations in Presenilin 1 (*PSEN1*), Presenilin 2 (*PSEN2*) or Amyloid precursor protein (*APP*), affecting APP processing, thus supporting the currently widely accepted “Aβ cascade hypothesis.” APP can be processed through the amyloidogenic or the non-amyloidogenic pathway, where the former leads to production of amyloid beta (Aβ) peptides (Figure 1). In AD there is an imbalance in terms of production and clearance of Aβ, and APP processing is shifted toward the amyloidogenic pathway. APP is initially cleaved by β-secretase, followed by γ-secretase, causing excessive production of Aβ peptides. These peptides can aggregate into insoluble Aβ plaques in the brain (Hardy and Higgins, 1992). Extracellular Aβ plaques is the main characteristic of AD, followed by the formation of intracellular neurofibrillary tangles (NFTs) caused by Tau hyperphosphorylation, and together these phenotypes are considered the neuropathological hallmarks of AD (Figure 2).

Although the pathology described above is still considered representative of the main AD features, the precise underlying mechanisms have yet to be identified. Other hypotheses have recently gained increasing interest. Those include organelle pathology such as Golgi and endoplasmic reticulum (ER) alterations, as well as neuroinflammation and the role of non-neuronal cells such as microglia and astrocytes in AD progression (Kinney et al., 2018). AD patients present with hyperactivation of the immune cells, most likely due to the inability to clear Aβ plaques, causing a chronic activated state with release of pro-inflammatory and neurotoxic factors, further promoting neurodegeneration (Figure 2). Several therapeutic approaches have been suggested in the search for an AD cure, targeting components in each of these hypotheses. However, most of these have failed in clinical trials, and treatment today is highly focused on symptom management (Liu et al., 2019). One of the challenges in terms of potential targets in AD treatment is that most molecules are not only implicated in AD but have important functions in other pathways, as seen with the approach of targeting the secretases involved in APP processing. While inhibition of these can reduce the amount of Aβ peptides, it will also impair Notch signaling, another γ-secretase substrate, essential in proper neurogenesis (Wolfe, 2012). The same can be seen for neuroinflammation. Microglia are crucial in maintaining a healthy homeostasis and defeating potential threats in the brain. On the other hand, chronic activation leads to pro-inflammatory and toxic effects. Thus, targeting these cells will have to be directed toward the detrimental effects, whilst maintaining the protective properties. Another issue, as previously mentioned, is that AD is often far progressed when diagnosed. Therefore, it is important to identify new preclinical biomarkers for AD together with potential new targets. Recent studies have suggested that glycosylation is implicated in AD, and considering the fact that most known AD-related molecules are either modified with glycans, or play a role in glycan regulation, glycobiology represent an interesting new insight into the understanding of AD, and a potential for new therapeutic approaches.

## Glycosylation

Biological information is transcribed from DNA to RNA, and can be further translated into proteins. Following translation, these proteins are often additionally modified. An important post-translational modification is the attachment of glycans to such proteins, providing important roles in recognition, energy metabolism, signaling and structure. Such modifications also provide the possibility of functional diverse products from a limited number of genes. Glycobiology is the study of the structure, biology, function and evolution of carbohydrates, or glycans and their associated aglycone (proteins, lipids or any other kind of molecule). All cells and a substantial number of molecules carry glycans, underlying their biological impact. Glycosylation is known to be the most common post-translational modification, and more than 50% of all proteins are glycosylated. Not surprisingly, it has therefore been suggested to be implicated in a number of diseases. In this review we will give a brief introduction to glycosylation, but for extended information we refer to textbooks such as *Essentials of Glycobiology* (Varki et al., 2015).

Carbohydrates can be classified as monosaccharides, oligosaccharides or polysaccharides. Monosaccharides are the simplest form of carbohydrates and can be linked together through glycosidic linkages to form the higher saccharide classes. Typically, oligosaccharides consist of less than 20 monosaccharides, while more complex structures are referred to as polysaccharides. The term glycan refers to carbohydrate structures that are attached to a protein, lipid or other molecule, forming a glycoconjugate. The complexity of a glycan can be highly variable depending on how many different types of monosaccharides it contains. Furthermore, glycans themselves can be modified by phosphorylation, acylation, methylation or sulfation, ensuring great diversity in terms of glycan function and mechanisms.

### Mechanisms and Major Types of Glycosylation

**FIGURE 3:**
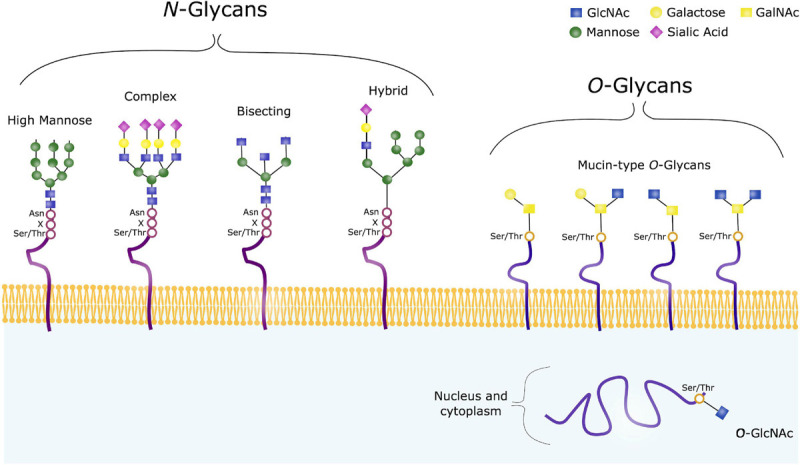
Common Glycan Structures. *N*-glycans consist of the same core consisting of GlcNAc and mannose, and can further be trimmed and modified from this core structure, often with galactose, fucose or sialic acids. High mannose, complex, hybrid and bisecting glycans are common types of such modified *N*-glycans. *O*-glycans on the other hand have varying core structures, consisting of either GalNAc or GlcNAc. *O*-GalNAc are referred to as Mucin-type *O*-glycans, and are usually found on membrane-bound, extracellular and secreted proteins. *O*-GalNAcylation is a complex form of *O*-glycosylation, and four common core structures are shown. *O*-GlcNAc on the other hand, are usually found on intracellular proteins.

Glycoconjugates are formed when sugar chains are added to proteins, lipids or other molecules, and in mammals 17 monosaccharides are commonly found in such glycan structures. Glycosylation can occur through various mechanisms, and includes addition of glycans to both proteins and lipids. There are two major types of protein glycosylation; the addition of *N*-linked glycans and *O*-linked glycans (Figure 3). *N*-glycosylation takes place in the ER and Golgi apparatus. Complex *O*-glycans are also mainly built in the Golgi, whereas *O*-GlcNAcylation occurs in the cytoplasm, nucleus and mitochondria.

*N*-glycosylation refers to addition of a *N*-acetylglucosamine (GlcNAc) to the peptide backbone, via a β-1N linkage to the nitrogen atom of an Asparagine (Asn). The initial process takes place in the ER, where the *N*-glycan precursor is synthesized from GlcNAc and Mannose. Mannose and glucose units are further added to form a glycan structure consisting of 14 sugars. The sugar chain is then transferred to a protein containing the sequence Asn-X-Serine (Ser)/Threonine (Thr). Following, the added glycan is trimmed by mannosidases and glucosidases in the ER, and further modified by glycosyltransferases in the Golgi apparatus to ensure glycan maturity (Reily et al., 2019).

When studying glycosylation, *N*-glycans, have the advantage that they are all comprised of a common core. This is however not the case in terms of *O*-glycosylation, a highly diverse form of glycosylation. *O*-glycans are covalently linked to a Ser/Thr, and in humans these are commonly GlcNAc or *N*-acetylgalactosamine (GalNAc). GalNAc-linked glycans are often referred to as mucin-type *O*-glycans, and the glycosylation process takes place in the *cis*-, medial- and *trans*-Golgi compartments. In the case of such complex *O*-glycosylation, trimming of existing sugar chain does not take place, unlike the pre- and post-processing steps seen in *N*-glycosylation. Modification of the *O*-glycan chains instead occur by specific glycosyltransferases with addition of galactose, GlcNAc, sialic acids or fucose. Such *O*-glycans are often found on extracellular and secreted glycoproteins. In contrast, intracellular glycoproteins often contain GlcNAc-linked glycans, where the biosynthesis usually takes place in the cytoplasm, regulated by *O*-linked GlcNAc transferase (OGT) and *O*-GlcNAcase (OGA). *O*-GlcNAc are attached to Thr and Ser residues, and no further sugars are added. This is a dynamic process where GlcNAc is rapidly added and removed from various protein substrates (Varki et al., 2015; Reily et al., 2019).

Increasing evidence indicate that both *N*-glycosylation and *O*-glycosylation are implicated in AD, emphasized by the fact that the two hallmark proteins APP and Tau carry both potential *N*-glycosylation and *O*-glycosylation sites.

### Regulation of Glycosylation

The process of glycosylation is mainly regulated by the key enzymes glucosidases and glycosyltransferases. Glucosidases function by hydrolyzing glycan linkages, whereas glycosyltransferases synthesize the glycan chain. Together they determine the structure of the glycan outcome. Mechanisms that regulate the gene expression of these enzymes, or the availability of specific substrates are therefore crucial in regulating the glycan composition of a cell.

**FIGURE 4:**
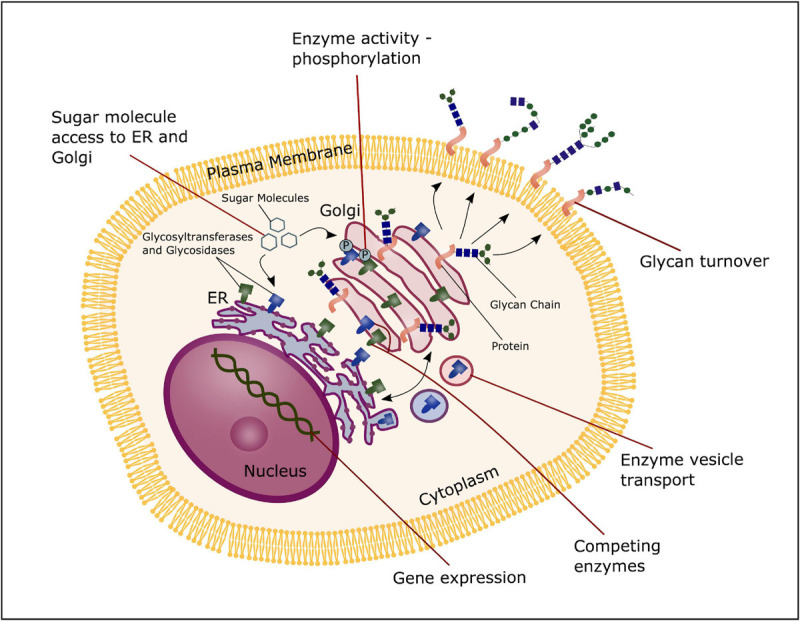
Glycosylation regulation. The process of glycosylation can be regulated at several stages; the transcription level of glycosyltransferases and glucosidases, transport and synthesis of sugar donors to the ER and Golgi, trafficking and localization of glycosyltransferases and glucosidases and substrate competition of these enzymes, phosphorylation of glycosyltransferase and glucosidase and total glycan turnover.

Glycosyltransferases and glucosidases are regulated both at a post-transcriptional and post-translational level, and multiple mechanisms can contribute in altering the expression, activity or structure of such enzymes. First of all, the formation of glycans can be regulated by the transcription levels of glycosidases and glycosyltransferases. Furthermore, mechanisms that control localization and trafficking of these enzymes is an important regulatory factor, as it influences the access to potential substrates. Transport and synthesis of sugar donors to both the Golgi and ER influence the availability of substrates, contributing to control of glycan formation. Additionally, the activity of glycosylation enzymes can potentially be modified through phosphorylation of the cytoplasmic tails (Ohtsubo and Marth, 2006), and enzymes competing for the same substrate will overall have an effect on the glycome of a given cell (Okada et al., 2015). Moreover, proteolysis that occur in the Golgi apparatus and total glycan turnover through endocytosis at the cell surface can impact glycan formation. Thus, multiple regulatory mechanisms are involved in modulation of glycan expression, contributing to the highly diverse pool of glycans and their function (Figure 4; Ohtsubo and Marth, 2006).

### Function of Glycans

Although glycobiology has been a field of many mysteries, research the past decades have revealed important functional roles of these highly diverse macromolecules, amongst others within the fields of cancer, infection and immunity, drug and pharmaceuticals, fertilization and blood types. Glycans are often present at the surface of cells, indicating a role in signaling and recognition. However, alongside the elaborate glycan diversity, comes a great range of functions. The biological role of these macromolecules can be broadly placed into four distinct categories. This includes structural and modulatory roles, extrinsic recognition, intrinsic recognition and host glycan mimicry (Table 1; Varki, 2017).

**TABLE 1:** Important glycan functions.

	**Glycan**	**Function**	
**Structural and Modulatory**	**Intrinsic Recognition**	**Extrinsic Recognition**	**Host mimicry**

Protection	Intracellular signaling	Immunity	Adaption of glycan surface
Stabilizer	Intracellular protein trafficking	Antigen uptake	Invasive pathogens
Tissue Integrity	intracellular Protein Folding	Viral, Bacterial, Fungal Adhesins	
Protein folding	SAMPs/DAMPs	PAMPs	
Storage center	Clearance receptors		

The modulatory and structural importance of glycans is underlined by the glycocalyx coating of eukaryotic cells and polysaccharide layer surrounding a number of prokaryotes, representing protective and stabilizing effects by providing a physical barrier. Furthermore, proteoglycans can contribute to maintenance of tissue integrity and overall organization, and ensure correct folding of newly synthesized proteins. Additionally, glycans modulate protein interactions, and in many cases ligand binding has been seen do be glycosylation-dependent. They can also function as a storage center for essential molecules, providing easy access and proper activity of such under specific conditions.

Glycans can act as specific ligands for cell-cell recognition and cell-matrix interactions, also known as intrinsic recognition. Intrinsic glycan functions can impact intracellular glycoprotein folding, trafficking and degradation, and play an important role in intracellular signaling. There is evidence that glycans can act as self-associated molecular patterns (SAMPs) to maintain a non-activated state of immune cells, or as danger-associated molecular patterns (DAMPs), overall ensuring cell homeostasis. In an extrinsic recognition matter, many glycoconjugates are acting as pathogen-associated molecular patterns (PAMPs) and facilitate antigen recognition and uptake, further emphasizing the immune-related role of glycans. These functions have not surprisingly led to host glycan mimicry, with pathogens adapting their glycan surfaces nearly identical to the host, thereby blocking recognition of underlying antigens, to increase their invasive capacities, providing yet another functionality of glycans (Varki et al., 2015; Varki, 2017).

The same glycans can also act differently within the same organism, and a glycan that serves a specific function in one tissue, can have a completely different role in another. Given the highly diverse functional properties of glycans, it is not surprising that altered glycosylation can play an important role in health and disease. Glycan dysregulation have been identified in a number of neurodegenerative disorders, and in AD the glycosylation profile of key disease regulators, such as APP, Tau, beta-secretase 1 (BACE1) and Nicastrin, have been shown to be altered.

## Glycosylation Alterations in Alzheimer’s Disease

**FIGURE 5:**
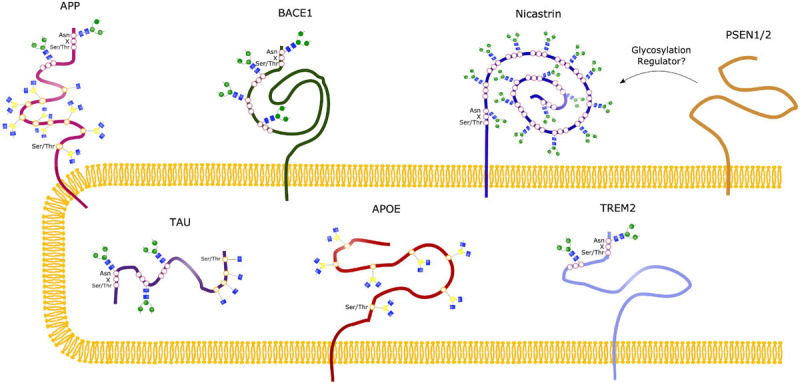
Glycosylation Sites of major AD molecules. Two *N*-glycosylation sites have been identified in APP, together with eight sites for *O*-glycosylation and sites for *O*-GlcNAcylation. Tau can be modified with *O*-GlcNAc at four potential sites, and only *O*-GlcNAcylation is observed in healthy brains. However, *N*-glycosylation of Tau have been observed in the case of AD, at three different sites. BACE1 only undergoes *N*-glycosylation, with four identified sites, whereas Nicastrin contains 16 potential *N*-glycan sites. No glycosylation sites have been observed in the presenilins (PSEN1/2), but studies suggest that these might play a regulatory role in glycosylation of other proteins, such as Nicastrin. The sporadic AD linked molecules APOE and TREM2 contains seven *O*-glycosylation sites and two *N*-glycosylation sites, respectively.

A lot of key proteins affected in AD are either glycosylated themselves or have an effect on the glycosylation of other proteins (Figure 5 and Table 2), supporting the hypothesis of a possible link between glycans and AD. AD patients present with an abnormal glycan profile, and several studies have shown altered glycosylation patterns in these patients. Increased levels of bisecting GlcNAc, which is a special *N*-glycan structure highly expressed in the brain, have been revealed in AD patients (Wang et al., 2019), together with an increased expression of the responsible converting enzyme GnT-III (Akasaka-Manya et al., 2010). Studies in neuroblastoma cells have shown an upregulation of GnT-III in response to Aβ treatment, and reduced Aβ load in GnT-III transfected cells, hypothesizing a protective effect of *N*-glycans, and upregulation of GnT-III as an adaptive response in AD brains (Akasaka-Manya et al., 2010). On the other hand, reports have shown that increased levels of bisecting *N*-glycans can promote AD pathogenesis by delaying BACE1 degradation. Loss of bisecting *N*-glycans have been shown to reduce Aβ generation and slow down AD progression in mice (Kizuka et al., 2015).

**TABLE 2:** Glycosylation in Alzheimer’s disease.

**Gene**	**Glycosylation Type**	**Comment**
APP	*N*-glycosylation *O*-glycosylation	Altered *N*-glycosylation can reduce APP processing and secretion. *O*-glycosylation reduce Aβ secretion
BACE1	*N*-glycosylation	Modifications with bisecting GlcNAc increase Aβ production, and is increased in AD.
Tau	*O*-GlcNAcylation *N*-glycosylation	*O*-glycosylation potentially play a protective role, decreased in AD patients. *N*-glycosylation of Tau is only observed in AD conditions, not in healthy controls.
Nicastrin	*N*-glycosylation	Immature and mature version, but effects of *N*-glycosylation is poorly understood
PSEN1	None	Potential regulatory role in glycosylation of other proteins
APOE	*O*-glycosylation	Alterations could potentially be linked to increased Aβ42 level
TREM2	*N*-glycosylation	Altered *N*-glycosylation could affect ligand binding properties and contribute to AD pathogenesis

Protein sialylation has been seen to be altered in AD, and a decrease in sialyltransferase (ST) has been identified in AD patients, observed in both serum (Maguire et al., 1994) and postmortem brain samples (Maguire and Breen, 1995). Sialylation is a terminal glycosylation process involving attachment of sialic acids to glycans/glycoproteins, catalyzed by ST. These sialic acids can play an important role in several cellular function by acting as recognition molecules, and *N*-acetylneuraminic acid (NeuAc) is the most abundant type of sialic acids in mammalian cells (Schedin-Weiss et al., 2014). Furthermore, altered sialylation profiles have been revealed in cerebrospinal fluid (CSF) of AD patients (Fodero et al., 2001), increasing the evidence toward an implicated sialylation and glycosylation process in AD pathogenesis. To elucidate how this implication contributes to the progression of AD, a number of studies have been conducted, looking into glycosylation of the most common AD-related proteins, which will be described in the following sections.

### APP

Amyloid precursor protein is the precursor protein that when cleaved by β-secretase in the amyloidogenic pathway form the toxic, aggregating Aβ peptides seen in AD patients. Both *N*-glycosylation and *O*-glycosylation sites have been identified in this protein, and both *N-* and *O*-glycosylated APP has been detected in the CSF of AD patients (Saito et al., 1993). APP has two potential sites for *N*-glycosylation, located at Asn467 and Asn496 (Påhlsson et al., 1992). APP *N*-glycans have been suggested to play a role in regulating the production of Aβ, as manipulation of such glycan structures have been observed to implicate APP transport and trafficking (Kizuka et al., 2017). Inhibiting *N*-glycan formation or maturity has been shown to reduce the secretion of APP in various models (Tienari et al., 1996; McFarlane et al., 1999, 2000). Furthermore, sialylation of APP-linked *N*-glycans seems to affect APP processing, whereas enhanced sialylation leads to an increase in APP secretion and Aβ production (Nakagawa et al., 2006), together with an increase in the bisecting GlcNAc level. Such alterations have been identified in relation to the familial AD causing mutations in the APP gene, such as the London and Swedish mutations, known to cause increased Aβ42/Aβ40 ratio, highlighting the potential link between glycosylation and AD pathogenesis (Schedin-Weiss et al., 2014).

A number of *O*-glycosylation sites, both for mucin-linked *O*-glycans and *O*-GlcNAc, have been identified in APP. *O*-GalNAcylation at sites Thr291, Thr292 and Thr576 have been observed in APP695 in Chinese hamster ovary cells (CHO) (Perdivara et al., 2009). Additionally, complex *O*-glycosylation sites in APP770 have been revealed at Ser597, Ser606, Ser662, Ser611, Ser680, Thr616, Thr635 and Thr635 in human CSF (Halim et al., 2011). Furthermore, APP has been seen to undergo *O*-GlcNAcylation (Griffith et al., 1995), and it has been suggested that such simple *O*-glycosylation can implicate the processing of APP, potentially by increasing non-amyloidogenic alpha-secretase (α-secretase) processing, the level of soluble APPα (sAPPα) and thus reducing the secretion of Aβ (Jacobsen and Iverfeldt, 2011). This has been investigated *in vitro* in HeLa cells transfected with the Swedish APP mutant. Inhibition of *O*-GlcNAcase in these transfected cells leads to increased levels of α-secretase products and decreased β-secretase processing. Increased *O* -GlcNacylation of APP can potentially affect the localization of the protein, promoting trafficking to the plasma membrane and decreasing endocytosis (Chun et al., 2015).

The link between *O*-GlcNAcylation and APP and Aβ is supported by *in vivo* studies in rats, showing that treatment with Aβ25-35 reduce the level *O*-GlcNAcylation in these rats. These findings correlate with up-regulation of glycogen synthase kinase-3-beta (GSK3β) and increased Tau phosphorylation (Lozano et al., 2017). Studies in 5xFAD mice models further highlight the relationship between *O*-GlcNAcylation and Aβ load, showing that inhibiting *O*-GlcNAcase, and thus enhancing *O*-GlcNAcylation, reduced the Aβ generation by lowering the activity of γ-secretase (Kim et al., 2013). In transgenic TAPP mice expressing both mutant human Tau and APP, *O*-GlcNAcase inhibition has also been thoroughly investigated, pointing toward a correlation between increased *O*-GlcNAcylation and reduced cognitive decline (Yuzwa et al., 2014). Although studies indicate that there is a potential link between *O*-GlcNAcylation of APP and the Aβ load in AD patients, it is important to bear in mind that other AD-related molecules are *O*-glycosylated, and could thus contribute to the effects seen by altered glycosylation (Schedin-Weiss et al., 2014).

### BACE1

In the amyloidogenic pathway, APP is initially processed by BACE1, producing soluble β-APP fragments (sAPPβ) and C99. C99 is further cleaved by γ-secretase to produce Aβ peptides of various lengths, with Aβ42 being the most toxic variant, prone to aggregate into the senile plaques’ characteristic for an AD brain. BACE1 can be *N*-glycosylated at four potential sites, and modifications at these sites have been suggested to affect the activity of the enzyme. On the other hand, no *O*-glycosylation sites have been detected in BACE1. It has been shown that the maturity and correct folding of BACE1 is highly dependent on *N*-glycan modifications, and that the number of such *N*-glycans correlates directly with folding, secretion rates and activity of the enzyme (Vanoni et al., 2008). BACE1 has been reported to be highly modified with bisecting GlcNAc, and as previously discussed, *in vivo* studies in mice show that knock-out of the *Mgat3*-gene, encoding the GnT-III enzyme, improve cognitive impairment and reduce Aβ deposition (Kizuka et al., 2015). *In vitro* studies with *Mgat-3* knock-out cells have revealed a shift in BACE1 localization toward late endosomes/lysosomes and thus leading to increased degradation. BACE1 localization in endosomal compartments is required for APP processing, and a shift toward lysosomal localization is suggested to be the cause of the drastic Aβ reduction observed in knock-out *Mgat3* studies (Tan and Evin, 2012). As bisecting GlcNAc on BACE1 is upregulated in AD patients, and could potentially also be linked to the oxidative stress observed in AD (Kizuka et al., 2016), inhibiting the GnT-III might be an interesting approach to reduce the Aβ load, indirectly targeting the BACE1 activity, yet circumventing the issues of adverse effects seen with BACE1 inhibitors (Kizuka et al., 2017).

BACE1 can also be linked to the altered protein sialylation seen in AD patients. One of the BACE1 substrates is known to be β-galactoside α2,6-sialyltransferase-1 (ST6GaI1), and BACE1 processing of this protein is required to generate the soluble ST form. BACE1 can thus affect sialylation of glycoproteins, and enhancement of these processes have been linked to increased APP secretion and Aβ production (Nakagawa et al., 2006).

### γ-Secretase (Nicastrin)

After APP is cleaved by β-secretase, Aβ peptides are generated through further processing of the C99 fragment via γ-secretase. This cleaving enzyme consists of four subunits; nicastrin, PSEN1 and PSEN2, Presenilin enhancer 2 (Pen-2) and Anterior pharynx-defective 1. Amongst these subunits, nicastrin has been suggested to be involved in γ-secretase substrate interactions (Bolduc et al., 2016), and it is the only subunit of γ-secretase that is known to be *N*-glycosylated, containing as much as 16 potential sites. Nicastrin contributes to γ-secretase activity by interacting with PSEN1 and PSEN2, the catalytic subunits of the enzyme, and complex glycosylation of nicastrin has been seen to be dependent on these presenilins (Yu et al., 2000). Two forms of nicastrin have been identified, an immature form with *N*-glycans not subjected to complex glycosylation, and a mature version carrying such complex *N*-glycans. It has been hypothesized that the maturity level is dependent on presenilins (Yang et al., 2002), and in PSEN1/2 knock-out cells the mature form of nicastrin cannot be identified, potentially due to impaired intracellular trafficking. However, the maturity level does not seem to affect its function (Herreman et al., 2003). Specific glycan structures related to nicastrin in neurons should be further elucidated to evaluate its role in nicastrin function.

### Presenilin 1

As previously stated, PSEN1 and 2 is the catalytic subunit of γ-secretase. Although no glycosylation sites have been identified, several studies have shown a regulatory role of presenilins in terms of glycosylation of other proteins, such as nicastrin discussed in the previous section. PSEN1 and 2 have also been observed to affect the glycosylation and sialylation of the neural cell adhesion molecule (NCAM), essential for brain function (Farquhar et al., 2003), and have an impact on the receptor Tyrosine-related kinase B, known to play a role in neural differentiation (Naruse et al., 1998). It has thus been suggested that the Presenilins can affect the glycosylation process in a number of proteins, either directly or by affecting the cellular location of proteins (Schedin-Weiss et al., 2014).

### Tau

Together with Aβ plaques, neurofibrillary tangles (NFTs) are considered the neuropathological hallmark of AD. These are found intracellularly and they are caused by abnormal hyperphosphorylation of the Microtubule-associated protein (MAP) Tau, which causes intraneuronal accumulation of paired helical filaments (PHF) eventually forming the NFTs (Iqbal et al., 2005). Tau is a cytosolic protein, and has been seen to undergo both *N*-glycosylation and *O*-GlcNAcylation, which is interesting given the fact that *N*-glycans are usually a modification seen with extracellular proteins, or at membrane-bound proteins extracellular domain. However, such *N*-glycosylation has been identified in AD patients, but not in healthy control brains, indicating an altered glycosylation process in such patients. These *N*-glycans have been identified at three sites; Asn359-Ile-Thr, Asn167-Ala-Thr and Asn410-Val-Ser (Sato et al., 2001; Liu et al., 2002; Losev et al., 2020), and have been proposed to affect the aggregation of Tau (Losev et al., 2019). Tau also undergoes *O*-GlcNAcylation, and four sites have been mapped to human Tau; Thr-123, Ser-208, Ser-400, Ser-409/Ser-412/Ser-413 (Zhu et al., 2014). In contrast to *N*-glycosylation, the level of *O*-GlcNAcylation has been observed to be decreased in AD brains compared to controls (Liu et al., 2004). It has thus been suggested that *O*-GlcNAcylation can play a protective role against the pathological hyperphosphorylation seen in AD. This is potentially due to the fact that *O*-GlcNAcylation and phosphorylation of Tau are two competing processes. Abnormal Tau phosphorylation could be caused by decreased *O*-GlcNAcylation, which again could be linked to the metabolic alterations/deficiencies observed in AD and other neurodegenerative disorders, underlying the potential role of glycosylation in disease progression (Liu et al., 2004; Zhu et al., 2014). The link between glycosylation, metabolic disorders and AD is further highlighted in a study investigating a mice model of Diabetes mellitus (DM). DM has been seen to increase the risk of cognitive dysfunction, and in these mice impaired learning and memory is seen together with obesity and hyperglycemia. These findings correlate with decreased *O*-GlcNAcylation and increased Tau phosphorylation, whereas hypoglycemic therapy improved these phenotypes, causing increased levels of *O*-GlcNAc transferase (Huang R. et al., 2020). Transgenic mice models of AD show the same trend, with an upregulation of Tau phosphorylation and reduced *O*-GlcNAcylation in the hippocampus, supporting the findings of an imbalance between *O*-GlcNAcylation and Tau phosphorylation (Gatta et al., 2016). This imbalance could potentially also affect the localization of Tau proteins (Lefebvre et al., 2003), and an overall affect on both function of Tau and formation of neurofibrillary tangles seen in AD patients (Shane Arnold et al., 1996). These findings highlight the potential of *O*-GlcNAcase inhibitors in treatment of AD, with the aim to prevent the pathological hyperphosphorylation of Tau (Yuzwa et al., 2014). However, increased insight into the specific mechanisms of *O*-GlcNAcylation of Tau is required to elucidate if the hyperphosphorylation in AD is in fact a cause or a consequence of decreased *O*-GlcNAcylation.

The altered glycosylation of the main AD-related molecules described above provides an evident implication of glycans in AD. Furthermore, organelle pathology such as ER stress and Golgi fragmentation have been observed in AD conditions, and could be a very interesting link to the glycan alterations seen in AD. A plausible hypothesis could be that the excessive Aβ accumulation and Tau phosphorylation cause a fragmentation of the Golgi, by causing inactivation of major Golgi proteins and cytoskeleton disruption, respectively (Joshi et al., 2015). Golgi fragmentation will most likely affect glycosylation, as this is the location for a majority of these processes, and could thus contribute to the glycan alterations seen in AD patients. Consequently, as the altered glycosylation can affect APP processing and Aβ load, together with Tau phosphorylation, it is not unlikely that it becomes a vicious cycle, enhancing each of these phenotypes. Elucidating the mechanisms behind this pathway would therefore be an interesting approach in the search for an AD cure.

In addition to the molecules described above, glycosylation has been observed to affect APOE, identified as a risk factor for developing sAD.

### APOE and Sporadic AD

ApoE is a cholesterol carrier important in transport of lipids and injury repair in the brain, and genetic variants within the *APOE* gene have been identified as the strongest genetic determinants of sAD risk (Liu et al., 2013). Three polymorphic alleles have been identified, with the ε4 allele being known to cause increased risk of developing AD. On the other hand, the ε2 allele has a protective role, whereas the most common ε3 variant has a neutral effect (Chartier-Harlin et al., 1994). Studies have shown a clear link between APOE genotype and Aβ, and although uncertainties in mode of action remains, the ε4 genotype has shown to increase both the intraneuronal Aβ accumulation and plaque deposition in postmortem AD brains (Yamazaki et al., 2019). Interestingly, APOE is as many other proteins modified by glycosylation. *O*-glycosylation of APOE was first identified at Thr194, and newly secreted APOE was found to be highly sialylated (Wernette-Hammond et al., 1989). More recently, a number of *O*-glycosylation sites have been identified, and the glycan profile has been seen to differ in the lipid-binding domain of APOE in CSF and plasma, indicating tissue specific glycoforms. Sialylated glycans are more abundant on the lipid-binding domain of CSF APOE, and could indicate that glycosylation plays a role in the flexibility of lipoprotein-binding (Flowers et al., 2020). APOE is known to interact with Aβ, and it has been suggested that the sialic moiety of APOE affects this interaction, thus being an important contributor in AD development (Sugano et al., 2008). Altered glycosylation profile of ApoE has been observed in a mouse model of Niemann-Pick Type C (NPC), a cholesterol-storage disorder that causes neurodegeneration, which shares some of the pathological mechanisms seen in AD, including Aβ deposition. In this study, they identified a potential link between changes in ApoE glycosylation and increased level of the toxic Aβ42 peptide (Chua et al., 2010).

Although the precise mechanisms of how APOE glycosylation contributes to AD pathogenesis have yet to be elucidated, there is clear indication that this process is implicated in the disease. Although the APOE ε4 allele is known as the strongest genetic risk factor for developing sAD, genome wide association studies (GWAS) have identified a number of genes that confers an increased risk of sAD. These are especially genes linked to the innate immune system, such as Triggering receptor expressed on myeloid cells-2 (*TREM2*), highlighting the role of microglia and neuroinflammation in AD (Efthymiou and Goate, 2017). Altered glycosylation pattern of TREM2 has been identified in AD, and provides an interesting link between glycosylation and neuroinflammation. This link is further emphasized by the fact that glycosylation plays an important role in cell-to-cell, as well as cell-environment interactions, which the immune system is highly dependent on. Neuroinflammation and glycosylation will therefore be elaborated in the following section.

## Neuroinflammation and Glycosylation

Until recently, AD research has been focused on neural pathology. However, increasing evidence support an important role of glial cells, such as microglia and astrocytes in disease pathogenesis. Neuroinflammation is a common characteristic seen in AD brains, and is now considered a highly interesting topic within the field of neurodegenerative disorders (Fakhoury, 2017). Especially the role of microglia has become evident, and the cells and genes related to the innate immune systems can be affected by glycans.

### Microglia and Glycosylation Alterations

Microglia are the resident macrophages of the brain, responsible for the innate immune system in the central nervous system (CNS). Although microglia only accounts for about 5% of the glial cell population in the cerebral cortex (Lawson et al., 1990), they play a vital role in terms of brain homeostasis and protection against potential threats (Mandrekar-Colucci and Landreth, 2012). In AD however, microglia have been observed to be associated with Aβ plaques, presenting with a proinflammatory phenotype. As they are unable to clear such plaques through phagocytosis, microglia in the condition of AD enters a state of chronic activation. These microglia release proinflammatory factors such as IL-1β, IL-6 and TNFα, which can be detrimental to surrounding neurons, further enhancing the neurodegenerative progression of the disease. Neuroinflammation can therefore be of large impact in AD pathogenesis (Mandrekar-Colucci and Landreth, 2012; Sarlus and Heneka, 2017). GWAS studies have revealed a number of genes related to the innate immune system as risk factors for developing AD, and lot of these genes are either highly or exclusively expressed by microglia, indicating an important microglial role in AD (Verheijen and Sleegers, 2018). Among the identified risk factors are the genes *TREM2* and *CD33* (Karch et al., 2012; Guerreiro et al., 2013). Altered glycosylation profile have been detected in TREM2 variants associated with sAD, providing a potential link between microglial neuroinflammation and glycosylation in AD progression. Furthermore, sialylation, which is altered in AD, have been suggested to play an important role in microglia-mediated neuroinflammation. CD33 is a sialic acid-binding receptor, within the sialic-acid-binding immunoglobulin-type lectin (SIGLEC) family, known to bind sialylated glycans, and plays an important role in microglia activation (Estus et al., 2019), highlighting the potential implication of glycosylation and sialylation in AD and other neurodegenerative disorders. Exploitation of these pathways could potentially serve as new target sites for therapeutic intervention (Puigdellívol et al., 2020).

### TREM2

TREM2 is expressed by microglia, and plays a role in modulating the inflammatory response and phagocytosis (Li and Zhang, 2018). Loss of TREM2 has been shown to reduce phagocytosis (Kawabori et al., 2015), whereas TREM2 overexpression has resulted in increased phagocytic activity (Takahashi et al., 2005). Genetic variations of TREM2 have been associated with increased risk of AD, and especially the identified missense mutation R47H have been linked to increased AD risk. Interestingly, studies have shown that the glycosylation pattern in this rare disease-associated TREM2 variant differs from the one seen in wild-type TREM2, having increased terminal glycosylation with complex oligosaccharides in the Golgi and decreased solubility, potentially affecting the function and ligand binding of the receptor, and in this way contribute to AD pathogenesis (Park et al., 2017). Furthermore, the TREM2 variants Y38C and T66M, linked with the early-onset disease Nasu-Hakola disease (NHD) display differences in the *N*-glycosylation profile, but varies from the profile seen within the R47H variant, potentially explaining the late-onset in AD versus the early-onset seen in NHD (Li and Zhang, 2018). Recently, studies have shown that TREM2 activity can be modulated by interactions with TMEM59. TMEM59 regulates complex glycosylation, secretion and cell surface expression of APP, with overexpression causing defects in glycan maturation (Ullrich et al., 2010). Furthermore, TMEM59 could play an important role in immunity, as downregulation has resulted in anti-inflammatory effects, potentially mediating microglia activity, functioning as a self-defense in microglia. TREM2 have been observed to interact with TMEM59, and potentially mediating the degradation of the protein. TREM2 deficiency cause impaired microglial survival and phagocytic activities in TREM2 knock-out mice, together with elevated levels of TMEM59. Downregulation of TMEM59 in these knock-out mice however, reversed such impairments, indicating a role of elevated TMEM59 in microglial defects. The fact that TMEM59 have an effect on both glycosylation and neuroinflammation in AD provides yet another potential connection between the two mechanisms (Liu et al., 2020).

### SIGLECs and Galectins

Lectins are carbohydrate binding proteins that can interact specifically with selected sugar structures, and which is a highly exploited feature used in glycoanalysis, as discussed in the following section (Van Damme, 2011). SIGLECs are lectins that play an important role in regulation of the immune response, and an important member of the SIGLEC family is CD33, also known as Siglec-3 (Crocker and Varki, 2001). Together with the evidence of altered sialylation in AD, such as decreased ST levels in these patients, desialylation of the microglial surface has been shown to be induced by activating stimuli such as LPS, Aβ and Tau, which can in turn enhance the complement receptor 3 (CR3) mediated phagocytosis of neurons (Allendorf et al., 2020). CD33 is expressed on microglia, and activation of this receptor can inhibit the phagocytic activity of microglia. On the other hand, sialylation of neurons can also contribute in inhibiting microglial phagocytosis of these neurons, protecting against neurodegeneration. When microglia are activated, such as in AD, they release a sialidase activity that can act by desialylating both neurons and microglia, promoting phagocytosis of neurons, and thus neurodegeneration. Microglial release of galectin-3 (Gal-3), another lectin member, binding to galactose residues (Dong et al., 2018), further activates microglia by Toll-like receptor 4 (TLR4) and TREM2 binding, as well as binding to desialylated neurons and promoting phagocytosis of such (Puigdellívol et al., 2020). These finding, together with the GWAS identification of CD33 as a risk factor for AD, underline the connection between altered sialylation of glycans and neuroinflammation in AD pathogenesis, providing new potential targets in preventing neuroinflammation and degeneration.

On an interesting note, inflammation in AD can be linked to the mitochondrial dysfunction observed in the disease, by increasing the production of reactive oxygen species (ROS), and such oxidative stress has been implicated in AD pathogenesis (Perry et al., 2002). Although glycosylation of mitochondrial proteins is a rather unexplored field, a number of such proteins have been suggested as targets for glycan modification, and may affect mitochondrial dysfunction and the oxidative stress response. The connection between mitochondrial dysfunction, glycosylation and inflammation has for example been reviewed in relation to Parkinson’s disease (PD) (Videira and Castro-Caldas, 2018). Furthermore, alterations of the glycosylation pattern of mitochondrial proteins have been observed in the cerebral cortex of a rat sAD model, indicating an implication of such processes also in AD (Yu et al., 2017), a potentially important future research area.

## Glycans as Biomarkers in Alzheimer’s Disease

Treatment of AD is challenging, partially because the disease is often far progressed when diagnosed. Pathologically, the course of AD starts decades before the clinical onset, further limiting the already restricted treatment options, emphasizing the need for new biomarkers and diagnostic tools. Even though the field of biomarkers in AD has advanced over the past few years, most studies have focused on markers for Aβ and Tau pathology, together with neurodegeneration, inflammation and synaptic deficiencies, as reviewed in Zetterberg and Bendlin (2020). Given the observations of aberrant glycome profiles in AD, these could potentially be used as new early stage biomarkers of AD. In this section tools used to identify and study glycans will be discussed, together with potential glycan biomarkers that can be beneficial in AD diagnosis and treatment strategies.

### Glycoanalysis

**FIGURE 6:**
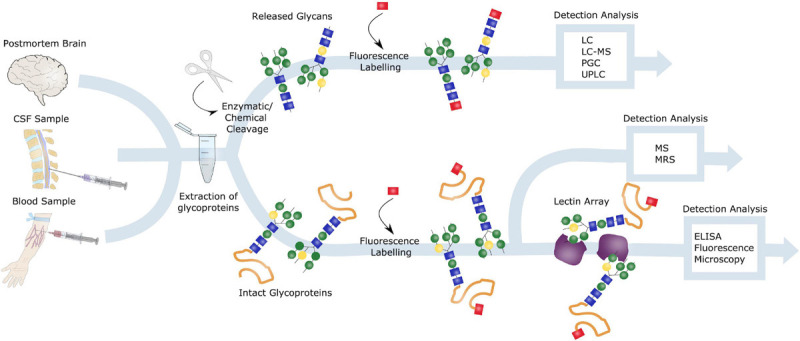
Glycoanalysis. Samples from postmortem brains, CSF and blood can all be obtained for glycoanalysis. Blood samples are especially of interest in terms of biomarker identification and AD diagnosis, due to the preferable less invasive procedure. Glycoproteins can be extracted from these samples, and either intact or released glycans can be investigated. Intact glycoproteins is commonly studied by lectin arrays and ELISA or fluorescence microscopy or by mass spectrometry (MS) or nuclear magnetic resonance spectrometry (MRS). To evaluate released glycoproteins, these can be either enzymatically or chemically cleaved, labeled and detected through liquid chromatography (LC), LC-MS, porous graphitic carbon chromatography (PGC) or ultra-high performance LC (UPLC).

There are several approaches to glycoanalysis (Figure 6), including investigation of intact glycoproteins and analysis of glycan structures after cleavage from their respective protein. In terms of studying intact glycoproteins as well as the localization of such, lectins are commonly used (Zou et al., 2017). Lectins recognizes and bind specific glycans and can be used for purification by methods such as fluorescence microscopy or enzyme-linked immunosorbent assay (ELISA) (Belický et al., 2016). Another highly exploited method for intact glycoprotein assessment is mass spectrometry (MS) (Domínguez-Vega et al., 2018), whereas nuclear magnetic resonance spectroscopy (MRS) can be used if a large amount of purified glycans is obtained (Zhang et al., 2016).

Removal of glycans, to investigate cleaved glycans, can be performed either enzymatically or chemically, by peptide *N*-glycosidase F (PNGase F) and hydrazinolysis, respectively. PNGase F cleaves *N*-linked glycans between the inner GlcNAc at the Asn residues, thus releasing the entire glycan structure. Furthermore, these released glycans can be analyzed using methods such as liquid chromatography (LC), porous graphitic carbon chromatography (PGC) or capillary electrophoresis. Prior to the LC analysis, glycans are usually fluorescently labeled. Often the advanced LC technique known as ultra-high performance LC (UPLC) is used to enhance the process (Regan et al., 2019).

To investigate the function of glycoproteins, inhibitors of the regulating enzymes glycosidases and glycosyltransferases are commonly used, and this technique is often exploited in cell- and animal models. Given that these enzymes are the main regulators of glycans, targeted inhibitors of these could make an interesting therapeutic approach to cope with the abnormal glycosylation observed in diseases (Rempel and Withers, 2008; Videira et al., 2018). Glycoanalysis is however more challenging in terms of *O*-linked glycans, due to the lack of a common core structure, and further optimization of tools for studying these is needed.

### Detection of AD Biomarkers and Diagnostic Properties

Although there are currently no validated glycan biomarkers for AD, several candidates are being investigated in ongoing studies. These biomarkers can be studied in various ways and can be broadly classified as biochemical (CSF-, blood sample), neuroanatomical (CT-, MRI scan), metabolic (PET, SPECT scan), genetic (e.g., APP/PSEN/APOE profile) and neuropsychological (e.g., Memory) (Wattamwar and Mathuranath, 2010). In terms of glycan biomarkers, studies are usually performed in samples from postmortem brains, CSF or blood (Figure 5). Identifying these biomarkers in serum from patients would be a great advantage in AD diagnostic, making the procedure as less invasive as possible.

Several studies have investigated the glycan profile in CSF from AD patients compared to healthy controls. One issue with this approach has been the fact that the CSF contains a rather low concentration of proteins, and a large volume is often required. However, recent studies have shown the possibility of performing CSF glycoanalysis with a very small amount of fluid (Cho et al., 2019). Increased levels of fucosylated and bisecting GlcNAc in AD CSF has been observed in several studies, which has also been confirmed in postmortem brains. Furthermore, the altered bisecting glycan profile correlates with CSF levels of phosphorylated and total Tau (Schedin-Weiss et al., 2020). A study of the CSF *N*-glycome in both mild cognitive impairment (MCI) and AD patients identifying 90 *N*-glycan structures by mass spectrometry revealed both a significant increase in bisected *N*-glycans together with a decrease in overall sialylation. Interestingly, the MCI patients that progressed to AD all showed such abnormalities in glycan profile, indicating that glycan alterations might precede the clinical onset of AD, highlighting their potentials as biomarkers for early AD diagnosis (Palmigiano et al., 2016). Many glycome studies have focused on *N*-glycans. However, the interplay between various glycosylation pathways have also been assessed both in brain tissue and serum. A simultaneous analysis of *N*- and *O*-glycomes revealed global alterations of protein glycosylation in both MCI and AD patients. Furthermore, the altered glycosylation pattern appeared to be region-specific in the brain, with *O*-GlcNAcylation observed to be decreased in the frontal lobe of AD brains, whereas it was increased in the hippocampus. Similar changes were observed in the serum of AD patients, which presented a unique glycol-fingerprint, that could be exploited to develop a new class of biomarkers for AD diagnosis, that thus could be obtained by a simple blood sample. This profile appears to be specific for AD, and differed from other neurodegenerative disorders (Frenkel-Pinter et al., 2017). Furthermore, blood plasma from AD patients has shown alterations in glycosylation of specific immunoglobulins (IgG), providing increased evidence of a correlation between neuroinflammation and glycosylation in AD (Lundström et al., 2014). Decreased ST activity and altered glycosylation of transferrin, an iron transport mediator, together with clusterin (CLU), an important player in debris clearance and apoptosis, both being genetically linked to AD, have been observed in AD blood samples (Maguire et al., 1994; Van Rensburg et al., 2004; Liang et al., 2015), making them potential blood biomarker candidates. An overview of potential glycan biomarkers in AD is presented in Table 3.

**TABLE 3:** Potential biomarkers for AD diagnosis.

**Biomarker**	**Alteration in AD**	**Detected in**
Bisecting GlcNAc	Increased	Postmortem brains, CSF and Serum
Fucosylated GlcNAc	Increased	Postmortem brains, CSF and Serum
ST activity	Decreased	Postmortem brains, CSF and Serum
Overall sialylation	Decreased	Postmortem brains, CSF and Serum
*O*-GlcNAcylation	Region-specific alteration	Postmortem brains
IgG	Decreased complex glycosylation and sialylation	Plasma
Transferrin	Decreased sialylation	CSF, serum
Clusterin	Decreased *N*-glycosylation	Plasma

## Glycans in Treatment of Alzheimer’s Disease

Today, only symptomatic treatment options exist for AD, and five FDA-approved prescription drugs are currently available. This includes the cholinesterase inhibitors Aricept (donepezil), Exelon (rivastigmine) and Razadyne (galantamine), and the *N*-methyl D-aspartate (NMDA) antagonist Namenda (memantine). Additionally, in moderate to severe cases of AD, Namzaric, a combination of donepezil and memantine, is commonly used. All of these can only manage symptoms of AD for a certain period of time, but cannot reverse or stop the disease progression, highlighting the need of new drug targets (Yiannopoulou and Papageorgiou, 2013). Several candidates are being investigated in ongoing clinical trials, and a lot of these are focusing on the amyloid-, tau- and neuroinflammatory hypotheses, as reviewed in Huang L. K. et al. (2020). Although many anti-amyloid trials are still being conducted, the number of such trials have decreased due to the constant failures, pointing toward a shift in treatment strategy, and need for novel targets.

Aberrant glycosylation can be identified in AD patients before the clinical disease onset. Thereby, re-establishing the glycosylation homeostasis could potentially be of interest in drug development. As previously described, glycosylation is mainly regulated by the action of glycosidases and glycotransferases. Modulating the function of these enzymes could thus be a potential therapeutic strategy (Wang et al., 2019). Furthermore, the increase in level of bisecting GlcNAc and decreased ST activity, together with the abnormal glycosylation linked to neuroinflammation presents potential new targets in AD treatment (Table 4).

**TABLE 4:** Potential therapeutic targets of glycosylation and drug candidates for AD.

**Drug Candidates**	**Mode of Action**
GnT-III inhibitors	Reduce the level of bisecting GlcNAc and Aβ deposition
*O*-GlcNAcase inhibitors	Reduce deglycosylation and phosphorylation of Tau
ST modulators	Increase sialylation, potentially preventing neuroinflammation
Sialidase modulators	Reduce desialylation, potentially preventing neuroinflammation
Gal-3 modulators	Reduce phagocytosis of neurons and Aβ production
CD33 modulators	Increase uptake and phagocytosis of Aβ deposits
GM1	Increase Aβ clearance and improve memory

### Novel Targets

#### Glycosyltransferase Inhibitors and Glycosidase Inhibitors

The activity of glycosyltransferases and glycosidases could be modulated directly or indirectly, and several methods can be used. One example is to inhibit the metabolism of glycan precursors or the glycan transport in the Golgi and ER. Other types of inhibition include blocking the addition of *N*-glycans to glycoproteins, for instance by using the inhibitor tunicamycin, or to use glycosidase or mannosidase inhibitors that prevents the formation of mature, complex glycoproteins. A suggested glycosyltransferase target in AD treatment is the GnT-III enzyme, responsible for the formation of bisecting glycans, observed to be upregulated in AD patients. GnT-III have been shown to reduce the Aβ deposition in mice, by acting on BACE1, without compromising the activity of the enzyme. BACE1 has several substrates besides APP, important for neural functions, making direct inhibition of the enzyme challenging and presented with a number of side effects and abnormalities in mouse models. Knocking out the GnT-III gene in such models has however not shown the same difficulties, and the mice remain healthy in these studies, indicating that the pathological effects of BACE1 could be selectively regulated by GnT-III glycosylation. GnT-III inhibitors could thus potentially be safer drug targets for AD treatment, circumventing the potential side effects seen with BACE1 inhibition (Kizuka and Taniguchi, 2018). Inhibition of glycosidases on the other hand, have been proven beneficial in terms of Tau pathology. Inhibiting the *O*-GlcNAcase, as shown with the use of compound Thiamet-G (Yuzwa et al., 2008), has reduced Tau phosphorylation, potentially by reducing the deglycosylation of the protein. Tau is modified by *O*-GlcNAc, and increasing the glycosylation could potentially compete with and thus reduce the pathological hyperphosphorylation of Tau seen in AD (Bojarová and Køen, 2009). A recent study further highlights this potential, presenting a promising new and selective OGA inhibitor; MK-8719. *In vivo* studies in a mouse model of tauopathy has shown that this compound can increase the level of *O*-GlcNAc and reduce pathologic Tau in the brains of these mice, protecting against brain atrophy (Wang et al., 2020). Inhibitors of the glycosylation enzymes could in this way target the pathological effects of AD related molecules, while maintaining the normal functions, thereby minimizing potential side effects. However, inhibition of OGA will most likely affect a great number of proteins, potentially causing chronic *O*-GlcNAc elevation. It would therefore be crucial to understand the exact mechanisms of such inhibitors and the consequences of elevated glycan levels, before implementing those in human clinical trials (Wang et al., 2020).

#### Sialylation Modulators: Siglecs and Galectins

As previously discussed, protein sialylation is implicated in AD, and decreased levels of ST has been identified in the serum of AD patients. ST are a large group of enzymes that attach sialic acids to glycoproteins, whereas the removal of such residues is performed by sialidases. High sialylation has been indicated to protect against neurodegeneration, and targeting the sialylation enzymes could therefore be of interest in drug development, either by increasing the sialylation process, or reducing desialylation. Microglia initiate sialidase activity when activated, that desialylate both microglia and neurons causing increased phagocytosis of surrounding neurons and in that way could contribute to neurodegeneration. Targeting sialidases could therefore contribute to prevent the neuroinflammation observed in AD.

Furthermore, lectins have been shown to be implicated in AD. Gal-3 is released by activated microglia, and contribute to phagocytosis of neurons, further promoting Aβ aggregation. Gal-3 could thus be another potential therapeutic target. The sialic acid binding microglial receptor CD33, that has been genetically linked to late onset AD, is yet another promising drug candidate. Single nucleotide polymorphisms in this gene have been shown to increase the risk of AD. On the other hand, reduced expression of the CD33 sialic acid-binding domain has been shown to confer protection against AD, introducing the potential beneficial use of CD33 inhibitors in AD treatment. Crystal structures of the CD33 protein have recently been studied, providing structural insights into the sialic binding sites and ligands that can increase phagocytosis and Aβ uptake, pointing out the possibility of therapeutic intervention at this binding site to promote Aβ clearance (Miles et al., 2019).

#### Gangliosides

In this review, the focus has been on glycosylation of proteins. However, glycosylation of lipids is another common process. Gangliosides are a type of glycosphingolipids containing one or more sialic acids, and play an important role in development and protection of the CNS. Ganglioside metabolism is reported to be associated with AD pathology, and changes in ganglioside profile have been observed in AD patients. Especially the GM1 ganglioside have been Investigated, and show neuroprotective roles, making it a potential therapeutic target in a number of neurodegenerative disorders, and such beneficial effects have already been described from clinical trials in the case of both stroke and PD (Magistretti et al., 2019). In AD, GM1 is observed to interact with Aβ, and in AD mouse models increased GM1 showed reduced Aβ accumulation and improved neuropathology (Bernardo et al., 2009). Beneficial effects of GM1 administration have also been observed in an AD rat model, improving memory deficits and spatial learning, potentially by mediating oxidative stress and lipid peroxidation (Yang et al., 2013). The protective actions of GM1 has been suggested to be initiated by increasing autophagy and promoting Aβ clearance by microglia (Yuyama et al., 2014; Dai et al., 2017). Further studies are however needed to confirm the GM1 mode of action in AD, establishing the therapeutic potential of the candidate in clinical use.

Research within neurological disorders can be challenging, due to the lack of sufficient models. Postmortem brains can only give insight into late stages of the diseases, whereas animal models often fall short in mimicking the precise human pathologies. A number of drugs that have proved to be potent in animal models, have failed to show the same efficacy in human trials. This issue is for instance evident with the potential drug target CD33, where differences in human and mouse properties of this protein is apparent (Bhattacherjee et al., 2019). Induced pluripotent stem cells could therefore provide an advantage in both disease modeling and therapeutic testing for glycosylation related disorders.

## Induced Pluripotent Stem Cell Modeling and Glycosylation

**FIGURE 7:**
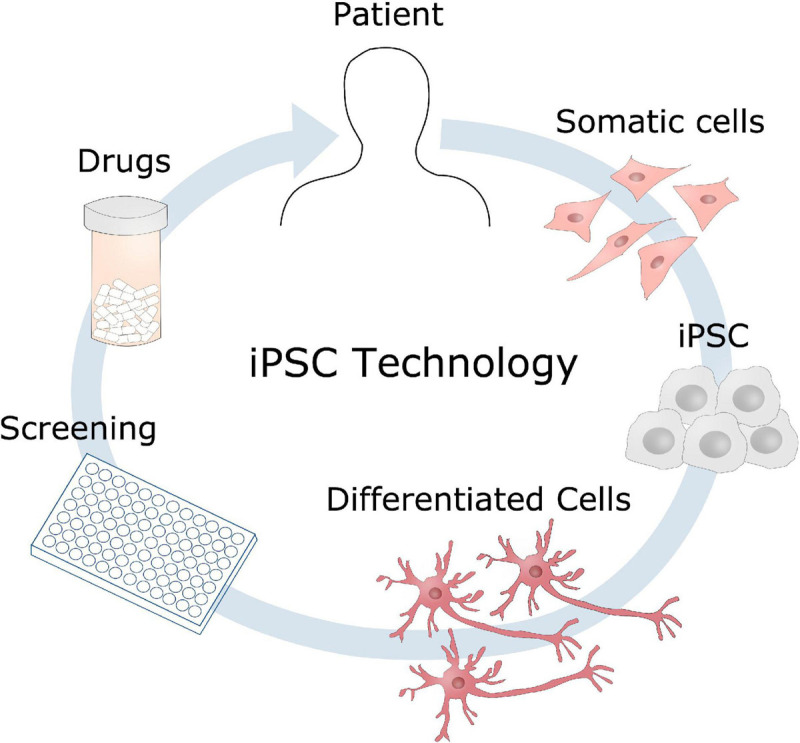
Induced pluripotent stem cells disease modeling. Somatic cells can be obtained from a patient, reprogrammed into induced pluripotent stem cells (iPSC), and further differentiated into the cell type of interest, such as neurons in the case of AD. These cell models could then be used for identifying disease mechanisms, drug screening to develop new therapeutic candidates.

The potential of reprogramming somatic cells into induced pluripotent stem cells (iPSC) revolutionized the scientific world, when introduced by Takahashi and Yamanaka (2006), Takahashi et al. (2007), providing a new platform for disease modeling and personalized medicine, whilst circumventing the ethical issues of embryonic stem cells. Since then, iPSC technology has been a widely exploited tool for modeling of a number of diseases and genetic disorders (Figure 7). iPSC allows study of cellular mechanisms within the same genetic background as the patient themselves, and the differentiation potential of such stem cells provides the possibility of investigating cell-type specific phenotypes, such as neurons and glial cells in AD. The implication of patient genetics can further be explored in these cell models using gene editing techniques such as CRISPR/Cas9, underlining the unique possibilities of iPSC in disease modeling (Hsu et al., 2014). The features of iPSC are being exploited in modeling of both glycosylation disorders as well as neurodegenerative disorders (Berger et al., 2016), and can be of great benefit to test the potential drug candidates identified in other model systems, on a human level. Implications of glycosylation that for instance have been observed in transgenic mice with mutations in APP and PSEN1 can be further investigated using iPSCs either from patients with the same mutations, or by introducing these mutations into healthy cells. This will be a great advantage to potentially validate disease phenotypes and therapeutic targets in a human model, which is an important step before translating it into clinical use. These cell models also provide a great test platform for potential drug candidates, which will evaluate the safety and potential side effects of drugs in a human matter.

## Concluding Remarks and Future Directions

Glycosylation affects both lipids and proteins, the latter accounts for the most common post-translational modification. Given the fact that more than 50% of all proteins are thought to be glycosylated, it is not surprising that this process could be implicated in a number of diseases. This is highlighted by the fact that major AD-related molecules such as APP, BACE1 and Tau are all modified by glycosylation and abnormalities of glycan pattern have been observed on several levels in AD patients. Furthermore, modulating the key enzymes glycotransferases and glycosidases have been shown to affect the Aβ load in AD, the main pathological hallmark of the disease, indicating that targeting the glycan balance could be a beneficial approach for AD intervention. In the scope of AD treatment, inhibitors of the APP processing molecules BACE1 and γ-secretase have been thoroughly investigated as drug candidates. However, such inhibitors will not only affect the pathogenic actions of these proteins, but will also implicate normal functions crucial for neural development. By targeting the glycan structures attached to AD-related molecules, and not the protein itself, one could potentially inhibit the harmful effects whilst maintaining the normal function of these proteins. With this approach one could potentially minimize side effects seen in current clinical trials.

Furthermore, altered glycosylation and sialylation has been observed in relation to the recently identified genetic AD risk factors TREM2 and CD33. These are both linked to microglia activity and neuroinflammation, presenting a possible link between neuroinflammation and glycosylation in AD.

Although studies have indicated that glycans could serve as new and reliable biomarkers for AD, and could be great targets for medical intervention, further research is necessary to confirm these potentials. One challenge is the fact that although modulators of the glycosylation process have been beneficial, precise mechanistic insights are still lacking, and due to the fact that multiple proteins are being modified in a similar matter, it is difficult to predict if the effects of treatments is caused by one or more proteins. Studying the potential link between glycosylation, ER stress and Golgi fragmentation could be an interesting new aspect in future AD research, to potentially elucidate these mechanisms. iPSC modeling provides a potential new platform for glycan research in neurodegenerative disorders, and could be a great addition accompanying the animal and postmortem studies. Whilst more knowledge is needed to confirm the role of glycosylation in AD pathogenesis, glycans remain as promising new biomarkers for early diagnosis and drug targets in AD treatment.

## Author Contributions

HH wrote the manuscript and prepared the figures. KF wrote and edited the manuscript. Both authors approved the final version. Both authors contributed to the article and approved the submitted version.

## Conflict of Interest

The authors declare that the research was conducted in the absence of any commercial or financial relationships that could be construed as a potential conflict of interest.
